# Structural Analysis of the Putative Succinyl-Diaminopimelic Acid Desuccinylase DapE from *Campylobacter jejuni*: Captopril-Mediated Structural Stabilization

**DOI:** 10.3390/cimb47121035

**Published:** 2025-12-12

**Authors:** Si Yeon Ahn, Young-Bong You, Han Byeol Oh, Min-Ah Park, Sung-il Yoon

**Affiliations:** Division of Biomedical Convergence & Department of Biomedical Science, Kangwon National University, Chuncheon 24341, Republic of Korea

**Keywords:** DapE, *Campylobacter jejuni*, structure, succinyl-diaminopimelic acid desuccinylase, captopril

## Abstract

DapE is a highly conserved bacterial enzyme that produces L,L-diaminopimelic acid in the *meso*-diaminopimelic acid and lysine synthesis pathway, which is essential for peptidoglycan formation in the cell wall. DapE has been recognized as a promising antibacterial drug target and can be inhibited by captopril. The pathogenic bacterium *Campylobacter jejuni* expresses a DapE ortholog, cjDapE. However, the structural basis underlying the enzymatic activity of cjDapE and its inhibition by captopril is unknown. Here, we report the crystal structures of cjDapE in complexes with Zn^2+^ and with both Zn^2+^ and captopril. cjDapE consists of a catalytic domain (CD) and a dimerization domain (DD). The CD harbors a pocket, which accommodates two Zn^2+^ ions in close proximity as the catalytic active site. cjDapE assembles into a dimer primarily using DD residues, with two DD loops largely disordered in the absence of captopril. Upon captopril binding, these loops become ordered and contribute to dimer stabilization by involving both DD and CD residues. Notably, captopril binding maintains cjDapE in an open conformation that is incompatible with catalytic activity. Our comparative structural analysis suggests that captopril inhibits cjDapE primarily via substrate competition.

## 1. Introduction

The *N*-succinyl-L,L-diaminopimelic acid desuccinylase DapE is a bacterial enzyme that catalytically hydrolyzes *N*-succinyl-L,L-diaminopimelic acid (SDAP) to L,L-diaminopimelic acid (DAP) and succinate [1]. DAP is ultimately converted to *meso*-diaminopimelic acid (*m*DAP) and to lysine via the enzymatic activities of DapF and LysA, respectively [2,3]. *m*DAP and lysine are essential for the formation of intact peptidoglycan in the cell walls of most Gram-negative and Gram-positive bacteria, respectively, through the crosslinking of peptidoglycan chains [4]. Therefore, DapE appears to be required for bacterial survival and growth. Indeed, deletion of the *dapE* gene in *Helicobacter pylori* and *Mycobacterium smegmatis* was lethal, even when lysine was supplemented in the growth medium [5,6].

Structural studies of DapE have revealed that DapE proteins from diverse bacterial species (*Haemophilus influenzae* DapE, hiDapE; *Neisseria meningitidis* DapE, nmDapE; *Acinetobacter baumannii* DapE, abDapE; *Enterococcus faecium* DapE, efDapE) commonly adopt a two-domain structure with an active-site pocket and assemble as dimers [7,8,9,10,11,12]. Each of the two pockets in the dimer can accommodate a pair of closely positioned Zn^2+^ ions that play a key role in catalysis. Moreover, the structural transition of the DapE dimer from an open conformation to a closed conformation has been proposed to be required for DapE-mediated SDAP hydrolysis [8,10].

Bacterial infectious diseases are typically treated with antibiotics, which target a limited number of biologically critical biomolecules, such as peptidoglycan-binding proteins, ribosomes, and dihydropteroate synthase [13]. In response to widespread antibiotic use, pathogenic bacteria have evolved diverse mechanisms to evade the detrimental effects of antibiotics. To overcome bacterial resistance to antibiotics, particularly multidrug resistance, a novel class of antibacterial drugs needs to be developed by identifying and targeting previously unexplored bacterial proteins. DapE has been proposed as a promising target for the development of antibacterial drugs with a novel mechanism of action because the *dapE* gene is essential for the survival and growth of most bacteria, is widely found as a conserved gene in pathogenic bacteria, including clinically significant multidrug-resistant species such as *Staphylococcus aureus* and *Acinetobacter baumannii*, and is absent in humans [14]. A previous screening study identified the thiol-containing compounds captopril and penicillamine as inhibitors of hiDapE, with reported *K*_i_ values of 1.8 μM and 4.6 μM, respectively [7,8,15,16]. Furthermore, captopril, which is clinically used to treat hypertension as an angiotensin-converting enzyme inhibitor, has been shown to exert antibacterial activity, highlighting its potential as an antibacterial drug [15]. A previous structural and biochemical analysis of the interaction between nmDapE and captopril suggested that captopril functions as a competitive inhibitor of nmDapE by inserting into the active site of nmDapE [7].

*Campylobacter jejuni* is a pathogenic bacterium that causes foodborne illness in humans [17,18]. Infection with *C. jejuni* can lead to enteritis, with symptoms of diarrhea, abdominal cramps, vomiting, and fever, and in rare cases, may progress to Guillain-Barré syndrome, an autoimmune neurological disorder [19]. Although *C. jejuni* infections are generally treated with the antibiotics ciprofloxacin and azithromycin, ciprofloxacin-resistant strains of *C. jejuni* are widespread, and resistance to azithromycin is steadily increasing, highlighting the need for a new type of anti-*C. jejuni* drugs [20,21].

*C. jejuni* also contains the *dapE* (*cj1048c*; NCBI accession number EAB5265329) gene, which encodes a 365-residue DapE protein (cjDapE) (Figure S1). However, structural studies have been reported for DapE proteins only from the phyla Pseudomonadota and Bacillota. Therefore, it is unclear whether cjDapE from the phylum Campylobacterota shares the structural features observed in other DapE proteins [7,8,9,10,11,12]. To gain insight into the structural mechanisms underlying the enzymatic activity of cjDapE and its response to captopril, we determined and analyzed the crystal structures of cjDapE and performed extensive comparative analysis of DapE structures.

## 2. Materials and Methods

### 2.1. Generation of the cjDapE Protein Expression Plasmid

The DNA that encodes cjDapE (residues 1–365) was amplified via PCR from the genomic DNA of *C. jejuni* (ATCC 33291) with Pfu polymerase and DNA primers containing the recognition sequence of the SalI or BamHI restriction enzyme at one end. The amplified DNA was treated with SalI and BamHI and subsequently ligated using T4 DNA ligase to a pET28b plasmid, which was designed to express a recombinant protein appended to a hexa-histidine (His_6_) tag and a thrombin cleavage site at the N-terminus [22]. The ligated plasmid was subsequently transformed into *Escherichia coli* DH5α cells, and the nucleotide sequence of the inserted DNA in the cjDapE expression plasmid was verified by DNA sequencing.

### 2.2. cjDapE Expression and Purification

For DapE overexpression, the cjDapE expression plasmid was transformed into *E. coli* BL21 (DE3) cells. The transformant cells were cultured in LB broth at 37 °C until the optical density of the culture at 600 nm reached 0.6–0.8. The cjDapE protein was overexpressed by further culturing the cells for 18 h at 18 °C in the presence of 1 mM isopropyl-β-D-1-thiogalactopyranoside. The cjDapE-containing cells were harvested via centrifugation at 4 °C and lysed by sonication in a solution containing 50 mM Tris, pH 8.0, 200 mM NaCl, 5 mM β-mercaptoethanol, and 1 mM phenylmethylsulfonyl fluoride on ice. The cell lysate was subjected to centrifugation at 4 °C to remove the cell debris, and the resulting supernatant was incubated with Ni-NTA resin (Qiagen, Germantown, MD, USA) for 1 h at 4 °C in the presence of 10 mM imidazole for the purification of the His_6_-tagged cjDapE protein via metal-immobilized affinity chromatography. The resin was harvested using an Econo-Column (Bio-Rad, Hercules, CA, USA) and treated stepwise with 50, 100, and 250 mM imidazole at room temperature for the elution of the tagged cjDapE protein. The eluted cjDapE protein was dialyzed against a solution containing 20 mM Tris, pH 8.0, and 5 mM β-mercaptoethanol and then treated with thrombin at 18 °C for 4 h to remove the His_6_ tag from cjDapE. The tag-free cjDapE protein was further purified via anion-exchange chromatography using a Mono Q 10/100 column (GE Healthcare, Hertfordshire, UK) at room temperature. The cjDapE protein was eluted from the column with a NaCl gradient (0–500 mM) in 20 mM Tris, pH 8.0, and 5 mM β-mercaptoethanol.

### 2.3. cjDapE Crystallization and X-Ray Diffraction

Protein crystallization was performed via a sitting-drop vapor-diffusion method at 18 °C by equilibrating a drop comprising 0.5 μL of a protein solution and 0.5 μL of a reservoir solution to 500 μL of the reservoir solution. The purified tag-free cjDapE protein was crystallized using a reservoir solution containing 40% PEG 300, 0.2 M calcium acetate, and 0.1 M sodium cacodylate, pH 6.5. The crystal of the cjDapE-captopril complex was obtained by cocrystallizing cjDapE and captopril at a molar ratio of 1:4 with a reservoir solution containing 34% PEG 400, 0.2 M magnesium chloride, and 0.1 M Tris, pH 9.0, and the resulting cjDapE-captopril crystal was soaked overnight in a solution containing 36% PEG 400, 0.2 M magnesium chloride, 0.1 M Tris, pH 9.0, and 10 mM captopril. cjDapE crystals were directly mounted for X-ray diffraction at the Pohang Accelerator Laboratory (PAL), beamline 7A. The collected X-ray diffraction data were indexed, integrated, merged, and scaled using the HKL2000 program (Table S1) [23].

### 2.4. cjDapE Structure Determination

The cjDapE structure was solved by molecular replacement using the AlphaFold3 model as a search model with the Phaser program [24,25]. The resulting molecular replacement solution was subjected to iterative cycles of manual model building and automatic refinement using the Coot and Phenix.refine programs, respectively, yielding the final structures of cjDapE and its complex with captopril (Table S1) [26,27]. The structures were analyzed by the Coot, Phenix, and CCP4 programs and visualized using the PyMOL program (version 3.1, https://www.pymol.org/).

### 2.5. Gel-Filtration Chromatography of cjDapE

The oligomeric state of cjDapE was investigated via gel-filtration chromatography. The tag-free cjDapE protein (100 μg) in a solution (300 µL) containing 20 mM Tris, pH 8.0, and 5 mM β-mercaptoethanol was loaded onto a Superdex 200 10/300 column (GE Healthcare) and eluted from the column using a solution containing 20 mM Tris, pH 8.0, 150 mM NaCl, and 5 mM β-mercaptoethanol. Protein elution was monitored by measuring the UV absorbance at 280 nm. Gel-filtration standards of 1, 17, 44, 158, and 670 kDa (Bio-Rad) were also loaded onto the column, and their elution volumes were used to generate a calibration curve correlating elution volume with the logarithm of molecular weight. This curve was then used to estimate the apparent molecular size of the cjDapE protein.

## 3. Results and Discussion

### 3.1. Overall Structure of cjDapE in Complex with Zn^2+^ Ions

**Figure 1:**
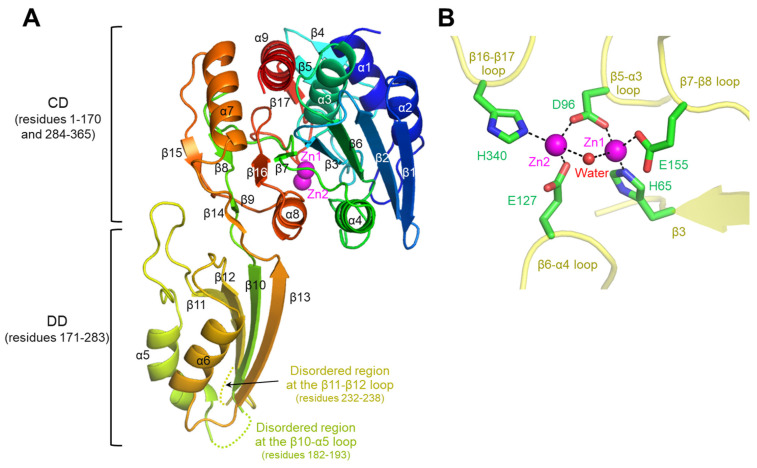
Overall structure of cjDapE_Zn_ and its interaction with Zn^2+^ ions. (**A**) Structure of a cjDapE_Zn_ monomer (rainbow ribbons; N-terminus, blue; C-terminus, red) with two Zn^2+^ ions (magenta spheres). The disordered regions at the β11-β12 loop and β10-α5 loop are represented by dotted lines. (**B**) Zn^2+^ ions (Zn1 and Zn2; magenta spheres) coordinated by cjDapE residues (green sticks) and a water molecule (red sphere) in the cjDapE_Zn_ structure (yellow transparent ribbons).

For the structural study of cjDapE, we expressed the full-length protein (residues 1–365) via the *E. coli* expression system and purified through metal-immobilized affinity chromatography and anion-exchange chromatography (Figure S2). The purified cjDapE protein was crystallized in the presence of PEG 300 at pH 6.5. The crystal structure of cjDapE was determined by molecular replacement and refined to an R_free_ value of 20.1% using X-ray diffraction data to a resolution of 1.95 Å (Table S1). The cjDapE structure contains cjDapE residues 1–181, 194–231, and 239–365, while the intervening regions (residues 182–193 and 232–238) are highly disordered (Figure 1A and Figure S1).

The cjDapE structure contains nine α-helices (α1-α9) and seventeen β-strands (β1-β17), adopting a two-domain architecture consisting of a catalytic domain (CD; residues 1–170 and 284–365) and a dimerization domain (DD; residues 171–283) (Figure 1A and Figure S1). The CD features a central six-stranded β-sheet (β1-β2-β6-β3-β7-β16), which is capped at one end by two small two-stranded β-sheets (β8-β15 and β9-β14) and is flanked on one face by two α-helices (α4 and α8) and on the other face by five α-helices (α1, α2, α3, α7, and α9) and a short three-stranded β-sheet (β4-β5-β17). This globular structure of the CD extends to the rod-shaped DD through two hinge loops. The DD adopts a two-layer α/β structure in which a four-stranded β-sheet (β11-β12-β10-β13) is decorated by the α5 and α6 helices on one face. Notably, the disordered regions of the cjDapE structure (residues 182–193 and 232–238) are located within the β10-α5 and β11-β12 loops at the distal end of the DD, far from the CD.

In the cjDapE structure, the CD harbors a pocket on the DD-facing side (Figure 1A). This pocket accommodates two discrete electron density peaks, each of which was assigned as a Zn^2+^ ion in the final structure based on the detection of zinc in the cjDapE crystal by X-ray fluorescence analysis and on analogous Zn^2+^ sites in orthologous structures. In the Zn^2+^-bound cjDapE structure (cjDapE_Zn_), the two Zn^2+^ ions (Zn1 and Zn2) are located in close proximity, with a distance of 3.7–3.8 Å, and are jointly coordinated by the side chain of the D96 residue and a water molecule (Figure 1B). The Zn1 and Zn2 ions are further coordinated by the side chains of the H65/E155 and E127/H340 residues, respectively. Zn1 is more deeply inserted into the pocket and displays higher electron density than Zn2. Consistently, in the structures of other DapE proteins, such as hiDapE and efDapE, Zn2 has been reported to show weaker or even absent electron density compared with Zn1 [9,12]. However, because cjDapE was crystallized in the presence of calcium acetate, we cannot exclude the possibility that the Zn2 site is partially occupied by Ca^2+^. Notably, this Zn^2+^-binding pocket is structurally analogous to the catalytic active site reported in hiDapE and abDapE [8,10].

### 3.2. cjDapE_Zn_ Dimerization

**Figure 2:**
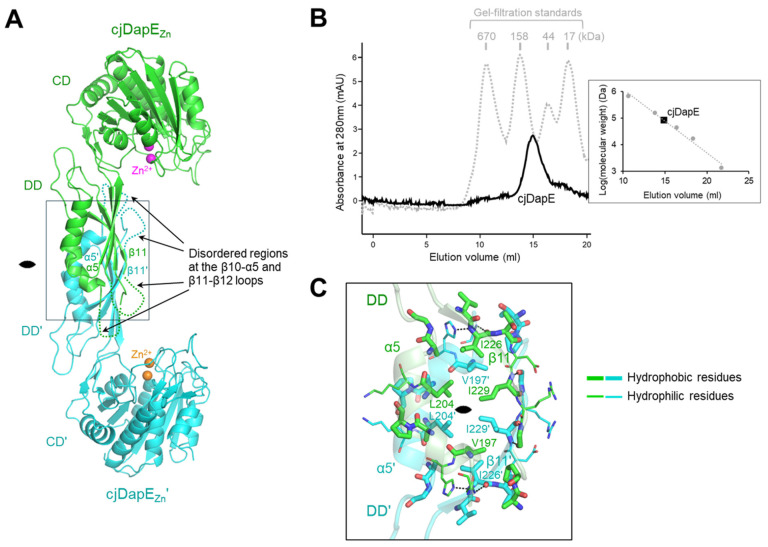
cjDapE_Zn_ dimerization. (**A**) Structure of a cjDapE_Zn_ dimer (cjDapE_Zn_ chain, green ribbons; cjDapE_Zn_′ chain, cyan ribbons; Zn^2+^, sphere). The disordered regions at the β11-β12 loop and β10-α5 loop are indicated by dotted lines. The 2-fold rotational pseudosymmetry is represented by a lens-shaped symbol. (**B**) Identification of a cjDapE dimer by gel-filtration chromatography. The elution profiles of cjDapE and gel-filtration standards are shown as black solid and gray dotted lines, respectively. A calibration curve (gray dotted line) generated from gel-filtration standards (gray circles), which plots elution volume against the logarithm of molecular weight, is presented in the right panel along with the elution volume of cjDapE (black square). (**C**) Dimerization interface in the cjDapE_Zn_ structure. Dimerization interface residues at the cjDapE_Zn_ (green transparent ribbons) and cjDapE_Zn_′ (cyan transparent ribbons) chains are shown as green and cyan sticks, respectively, with the hydrophobic and hydrophilic residues represented by thick and thin sticks, respectively. The figure is an enlarged view of the rectangle in (**A**).

In the cjDapE_Zn_ crystal, cjDapE_Zn_ adopts a dimer structure via noncrystallographic twofold rotational symmetry within the asymmetric unit (Figure 2A). The two cjDapE_Zn_ chains exhibit essentially identical structures with a root-mean-square deviation (RMSD) value of 0.40 Å. cjDapE_Zn_ dimerization is driven solely by the DD, without the contribution of the CD. The DD and DD′ occupy the center of the dimer, engaging the two cjDapE_Zn_ chains, whereas the CD and CD′ project from the DD-DD′ core, forming the two ends of the dimer (prime denotes the second chain). Therefore, the Zn^2+^-binding pocket from one cjDapE_Zn_ chain is oriented toward the dimer core, facing the disordered regions of the β10-α5 and β11-β12 loops at the opposing cjDapE_Zn_ chain.

The dimeric state of cjDapE_Zn_ was confirmed by gel-filtration chromatography (Figure 2B). In gel-filtration chromatography, the purified DapE protein, with a calculated molecular weight of 41.0 kDa, eluted as a single peak between the 44 and 158 kDa standards, yielding an apparent molecular size of 79.8 kDa. This analysis indicates that cjDapE_Zn_ also forms a stable dimer in solution, as observed in the crystal structure.

cjDapE_Zn_ dimerizes using eighteen residues from the α5 helix, the β11 strand, and their adjacent loops (β10-α5, α5-β11, and β11-β12 loops), burying a surface area of ~810 Å^2^ (Figure 2A,C). The β11 strand and its C-terminal β11-β12 loop in the DD align antiparallel to and interact with their counterparts in the opposing DD′, forming reciprocal main-chain hydrogen bonds (I229-G231′ and G231-I229′) in the middle. This β11-β11′ interaction allows the β-sheet of the DD to merge with that of the DD′, forming a continuous β-sheet across the cjDaeE dimer. The α5 helix and its adjacent β10-α5 and α5-β11 loops also form antiparallel interactions with the corresponding regions of the DD′. The central region of the cjDapE_Zn_ dimerization interface is dominated by hydrophobic interactions, which are mediated primarily by residues V197, L204, I226, and I229, whereas hydrophilic interactions are localized mainly at the periphery of the interface.

### 3.3. Interaction of cjDapE with Captopril and Its Contribution to Structural Stabilization

**Figure 3:**
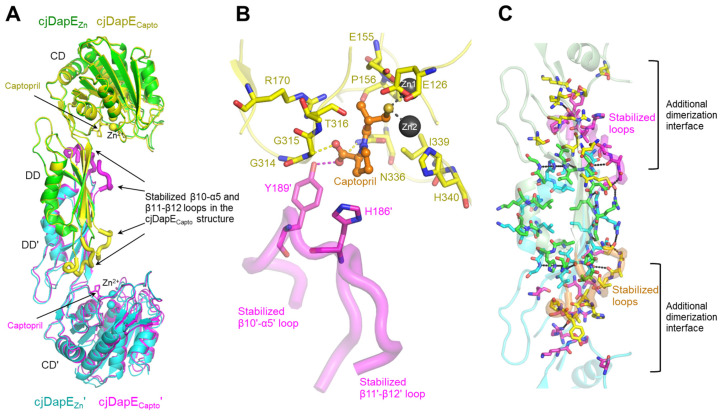
cjDapE_Capto_ structure and captopril binding-mediated structural stabilization. (**A**) Structural comparison of cjDapE_Zn_ (green or cyan ribbons) and cjDapE_Capto_ (yellow or magenta ribbons) dimers. Zn^2+^ ions and captopril are represented by spheres and sticks, respectively. The regions of cjDapE_Capto_ at the β10-α5 and β11-β12 loops stabilized by captopril binding are highlighted by thick coils. (**B**) Interaction of captopril (orange ball-and-stick model) with cjDapE residues (yellow or magenta sticks) in the cjDapE_Capto_ structure (cjDapE_Capto_ chain, yellow transparent ribbons; β10′-α5′ and β11′-β12′ loops stabilized by captopril binding, magenta transparent coils). (**C**) Extended dimerization interface in the cjDapE_Capto_ structure. Captopril binding to cjDapE (pale green or cyan transparent ribbons) induces the stabilization of the β10-α5 and β11-β12 loops (orange or magenta transparent thick coils), extending the dimerization interface of cjDapE. The dimerization interface residues observed in both the cjDapE_Zn_ and cjDapE_Capto_ structures are shown as green or cyan sticks, and those observed only in the cjDapE_Capto_ structure are depicted as yellow or magenta sticks.

To elucidate the structural mechanism whereby captopril recognizes DapE and inhibits the enzymatic activity of DapE, we determined the crystal structure of cjDapE in complex with Zn^2+^ and captopril (cjDapE_Capto_) at a resolution of 2.45 Å, with an R_free_ of 22.4% (Table S1). The cjDapE_Capto_ structure contains nine α-helices and seventeen β-strands, consists of the CD and DD, and features a pocket with two Zn^2+^ ions, as observed for the cjDapE_Zn_ structure (Figure 3A). Furthermore, cjDapE_Capto_ dimerizes in an organization essentially identical to that of cjDapE_Zn_, with an RMSD value of 0.34 Å. Interestingly, the central regions of the β10-α5 and β11-β12 loops, which are disordered in the cjDapE_Zn_ structure, become stabilized and structurally resolved in the cjDapE_Capto_ structure (Figure 3A). These regions are located at the distal end of the DD and are oriented toward the CD of the opposing chain.

In the cjDapE_Capto_ structure, a captopril molecule is observed in the pocket of cjDapE near Zn^2+^ ions (Figure 3B and Figure S3). Captopril consists of a proline moiety and a thiol-containing tail. The proline moiety of captopril points outward from the pocket, whereas the thiol moiety of captopril is deeply inserted into the pocket and interacts with the two Zn^2+^ ions, replacing the Zn^2+^-coordinating water molecule observed in the cjDapE_Zn_ structure. Because this water molecule was proposed to function as a hydroxide nucleophile that mediates SDAP hydrolysis via nucleophilic attack, its abstraction from the active site by captopril represents one of the mechanisms underlying the inhibitory effect of captopril.

In the cjDapE_Capto_ structure, captopril recognizes ten pocket residues from the cjDapE_Capto_ chain, in addition to the Zn^2+^ ions (Figure 3B). Particularly, the carboxyl group of the proline moiety forms hydrogen bonds with residues G315 and N336. Notably, the proline moiety of captopril further contacts Y189′ and H186′ from the DD’ of the opposing chain, forming a hydrogen bond between its carboxyl group and the hydroxyl group of Y189′. Because Y189′ and H186′ are located in the middle of the stabilized β10′-α5′ loop, their interactions with captopril likely provide the driving force for stabilizing the β10′-α5′ loop (and even the neighboring β11′-β12′ loop) when captopril binds. It remains unclear whether this captopril-mediated structural stabilization arises from direct interactions between cjDapE and captopril or from indirect allosteric effects. However, this stabilization is unlikely to result from a crystallographic artifact because the β10-α5 and β11-β12 loops do not participate in crystal packing, and cjDapE_Capto_ and cjDapE_Zn_ were crystallized in the same space group with similar unit-cell parameters.

Notably, in the cjDapE_Capto_ dimer structure, the captopril-stabilized β10-α5 and β11-β12 loops extensively interact with CD’ and DD’ residues from the opposing chain at the β9′-β10′ loop, β10′ strand, α5′-β11′ loop, β12′ strand, β12′-α6′ loop, β15′-α8′ loop, and β16′-β17′ loop (Figure 3C). Consequently, the dimerization interface expands bidirectionally toward the CD and CD′ and involves residues from the CD and CD’ as well as DD and DD’ residues, resulting in a marked increase in the buried surface area to ~1550 Å^2^ upon dimerization and thereby reinforcing cjDapE dimer formation.

### 3.4. Open, Inactive Conformation of the Captopril-Bound cjDapE Structure

**Figure 4:**
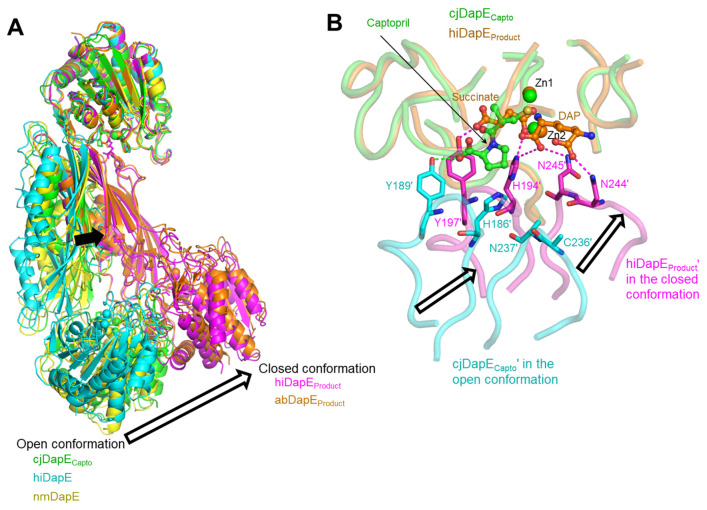
Open, inactive conformation of cjDapE_Capto_. (**A**) Open conformation in product-free DapE structures (cjDapE_Capto_, green ribbons; hiDapE, PDB ID 3IC1, cyan ribbons; nmDapE, PDB ID 4O23, yellow ribbons) and closed conformation in product-bound DapE structures (hiDapE_Product_, PDB ID 5VO3, magenta ribbons; abDapE_Product_, PDB ID 8F8O, orange ribbons). These structures are superimposed based on one CD. Structural differences between the open and closed conformations are highlighted by arrows. (**B**) Captopril-mediated stabilization of cjDapE_Capto_ in an open conformation compared with the closed conformation of hiDapE_Product_. The hiDapE_Product_ dimer structure (PDB ID 5VO3; succinate and DAP products, orange ball-and-stick models; Zn^2+^, orange sphere; hiDapE_Product_ chain, orange transparent coils; hiDapE_Product_′ chain, magenta transparent coils) is superimposed on the cjDapE_Capto_ dimer structure (captopril, green ball-and-stick model; Zn^2+^, green sphere; cjDapE_Capto_ chain, green transparent coils; cjDapE_Capto_′ chain, cyan transparent coils) based on one CD. The hiDapE_Product_′ residues that form hydrogen bonds (magenta dotted lines) with the products are depicted as magenta sticks, and their corresponding residues in the cjDapE_Capto_′ chain are shown as cyan sticks, with the hydrogen bond of captopril with Y189′ indicated by green dotted lines.

cjDapE exhibits the highest structural homology with its orthologs based on a search in the protein structure comparison server Dali [28]. The cjDapE_Capto_ structure shows RMSD values of 1.6–5.5 Å over 327–361 aligned residues when compared with the orthologous structures of hiDapE, nmDapE, abDapE, and efDapE, all of which adopt a two-domain architecture with a pocket and assemble into a dimer, as observed for the cjDapE_Capto_ structure (Figure 4A) [7,8,9,10,11,12]. In terms of interdomain organization and dimeric architecture, DapE structures can be broadly classified into two groups: an open conformation and a closed conformation (Figure 4A). Compared with the cjDapE_Capto_ structure, the hiDapE and abDapE structures in complex with both the product and Zn^2+^ adopt a substantially more closed conformation [8,10]. In contrast, the cjDapE_Capto_ structure shows relatively small deviations from other orthologous structures representing the apo state or complexes with ligands other than products [7,9,11,12]. These product-free structures, including cjDapE_Capto_, can be defined as adopting an open conformation.

The conversion of the open conformation to the closed conformation is key to DapE-mediated catalysis, as suggested by the structural differences between the product-bound and product-free forms of hiDapE [10]. In the product-bound hiDapE structure (hiDapE_Product_), one of the two products, succinate, is inserted into the active-site pocket, and the other product, DAP, is positioned adjacent to succinate and extends from the pocket toward the exterior (Figure 4B). The two products engage in extensive interactions with residues from the hiDapE_Product_′ DD′ as well as the hiDapE_Product_ CD. In particular, interactions with the hiDapE_Product_′ DD′ occur at residues within the β10′-α5′ and β11′-β12′ loops. Succinate forms hydrogen bonds with H194′ and Y197′ at the β10′-α5′ loop, and DAP mediates hydrogen bonding with H194′ and N244′/N245′ at the β10′-α5′ and β11′-β12′ loops, respectively. As a result, the DD′ moves closer to the CD upon product binding, allowing the hiDapE_Product_ structure to adopt the closed conformation. Notably, in the closed conformation, H194′ is located near both products and Zn^2+^ and was previously proposed to form an oxyanion hole with Zn^2+^ as a catalytically critical residue [10]. Therefore, the closed and open conformations represent catalytically active and inactive states, respectively.

Consistent with the DapE-inhibiting property of captopril, the cjDapE_Capto_ dimer adopts the open, inactive conformation rather than the closed, active conformation. Although the proline moiety of captopril from the cjDapE_Capto_ structure partially overlaps with succinate from the hiDapE_Product_ structure, it protrudes further from the pocket compared with succinate, displacing the hydrogen-bonded cjDapE_Capto_′ Y189′ of the β10′-α5′ loop away from the position of its corresponding residue (Y197′) in hiDapE_Product_′. Furthermore, the hydrogen bonds between the products and the hiDapE_Product_′ DD′ residues H194′, N244′, and N245′ of the β10′-α5′ and β11′-β12′ loops, which stabilize the hiDapE_Product_′ structure in the closed conformation, are absent in the cjDapE_Capto_ structure due to the lack of additional hydrogen bonding groups in captopril. As a result, the β10′-α5′ and β11′-β12′ loops of the cjDapE_Capto_′ DD′ are located farther from the CD in an open conformation than those of the hiDapE_Product_′ DD′ in the closed conformation, preventing the putative catalytic residue H186′, corresponding to hiDapE_Product_′ H194′, from attaining a catalytically competent position. Therefore, captopril-bound cjDapE is stabilized in an inactive, open conformation.

To elucidate the inhibitory mechanism of captopril, the cjDapE_Capto_ structure was overlaid onto the closed, active structure of hiDapE (hiDapE_Product_) using one CD as a reference (Figure 4) [10]. The thiol tail of captopril in the cjDapE_Capto_ structure overlaps with the succinate product in the hiDapE_Product_ structure. Moreover, the captopril-binding residues of cjDapE are identical in residue type to the succinate-binding residues of hiDapE and generally adopt similar conformations and positions to those in the hiDapE_Product_ structure (Figure S3). These structural observations suggest that captopril inhibits substrate binding by directly competing with the succinyl group of the SDAP substrate for the active-site pocket (Figure 4B).

### 3.5. Similar but Distinct Modes of Captopril Binding Between cjDapE and nmDapE

**Figure 5:**
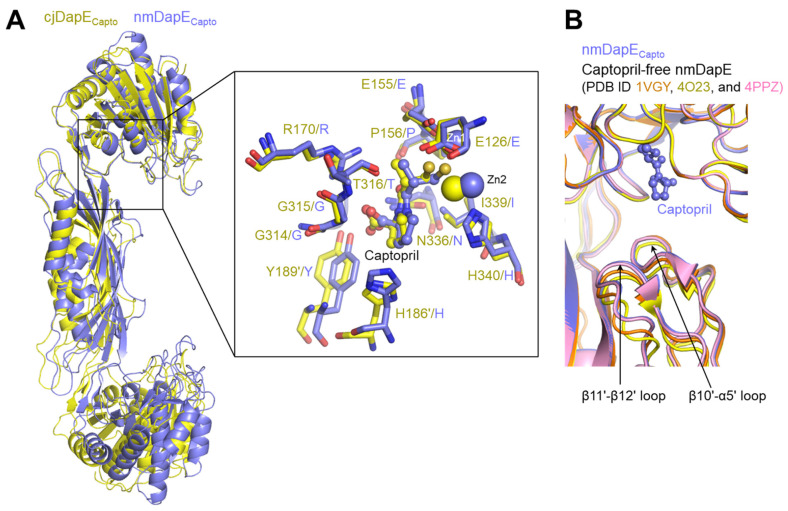
Similar but distinct captopril-binding modes between cjDapE and nmDapE. (**A**) Overlaid structures of cjDapE_Capto_ (yellow ribbons) and nmDapE_Capto_ (light blue ribbons) showing their overall similar captopril-binding modes. In the inset, the captopril-binding residues of cjDapE_Capto_ (yellow) and nmDapE_Capto_ (light blue) are shown as sticks, together with Zn^2+^ ions and captopril, and are labeled before and after the slash, respectively. (**B**) Captopril-independent stabilization of the nmDapE regions corresponding to the β10′-α5′ loop and β11′-β12′ loop of cjDapE. The nmDapE_Capto_ structure (PDB ID 4PQA) is displayed as light blue ribbons with captopril shown as a light blue ball-and-stick model. The captopril-free structures of nmDapE are depicted as orange (PDB ID 1VGY), yellow (PDB ID 4O23), and pink (PDB ID 4PPZ) ribbons.

Another captopril-bound DapE structure has been reported for nmDapE [7]. In the nmDapE-captopril complex structure (nmDapE_Capto_), captopril is inserted into the active site and retains nmDapE in an open conformation, similar to the cjDapE_Capto_ structure (Figure 5A). Moreover, the thiol group of captopril is positioned between the two Zn^2+^ ions, replacing the active-site water molecule observed in the captopril-free structure, consistent with the binding mode in cjDapE. Furthermore, captopril-binding residues from both chains in the dimer are generally conserved in sequence and structure between nmDapE and cjDapE. Therefore, the captopril-mediated inhibition mechanism identified for cjDapE is also employed in the inhibition of nmDapE, implying that this inhibitory strategy is conserved across diverse bacterial species.

Notably, nmDapE does not undergo any substantial structural changes upon captopril binding (Figure 5B) [7]. The regions corresponding to the β10-α5 and β11-β12 loops of cjDapE are already stabilized even before captopril binding and display nearly identical structures regardless of captopril binding. This captopril-independent stabilization in nmDapE stands in stark contrast to cjDapE, in which these regions become stabilized only after captopril binding. Therefore, the captopril-mediated stabilization of the β10-α5 and β11-β12 loops is not observed in nmDapE and seems to be specific to cjDapE.

In conclusion, we determined and comparatively analyzed the crystal structures of cjDapE in two forms: one bound to Zn^2+^ ions and the other bound to both Zn^2+^ ions and captopril. cjDapE forms a two-domain dimeric architecture with each monomer containing a pocket that coordinates two Zn^2+^ ions. Captopril occupies the pocket near the two Zn^2+^ ions and orders the β10-α5 and β11-β12 loops in a cjDapE-specific manner, stabilizing the dimer in an open, inactive conformation. Our comparative structural analysis indicates that captopril inhibits the enzymatic activity of DapE through a competitive binding mechanism.

## Data Availability

The atomic coordinates and the structure factors for cjDapE (PDB ID 9X8G and 9X75) have been deposited in the Protein Data Bank (www.rcsb.org). Further inquiries can be directed to the corresponding author.

## Supplementary Materials

The following supporting information can be downloaded at: https://www.mdpi.com/article/10.3390/cimb47121035/s1.

## Abbreviations

The following abbreviations are used in this manuscript:

abDapE	*Acinetobacter baumannii* DapE
cjDapE	*Campylobacter jejuni* DapE
cjDapE_Capto_	*Campylobacter jejuni* DapE in complex with Zn^2+^ and captopril
cjDapE_Zn_	Zn^2+^-bound *Campylobacter jejuni* DapE
DAP	L,L-diaminopimelic acid
efDapE	*Enterococcus faecium* DapE
His_6_	hexa-histidine
hiDapE	*Haemophilus influenzae* DapE
hiDapE_Product_	product-bound *Haemophilus influenzae* DapE
*m*DAP	*meso*-diaminopimelic acid
nmDapE	*Neisseria meningitidis* DapE
nmDapE_Capto_	*Neisseria meningitidis* DapE-captopril complex
PAL	Pohang Accelerator Laboratory
SDAP	*N*-succinyl-L,L-diaminopimelic acid
