# Supplementary material for: Structural Analysis of the Putative Succinyl-Diaminopimelic Acid Desuccinylase DapE from Campylobacter jejuni: Captopril-Mediated Structural Stabilization
Source: Curr Issues Mol Biol. 2025 Dec 12;47(12):1035. doi: 10.3390/cimb47121035 (PMC12731327; doi:10.3390/cimb47121035)
Supplement: Supplementary file 1 [file cimb-47-01035-s001.zip › cimb-4013436-supplementary.pdf]

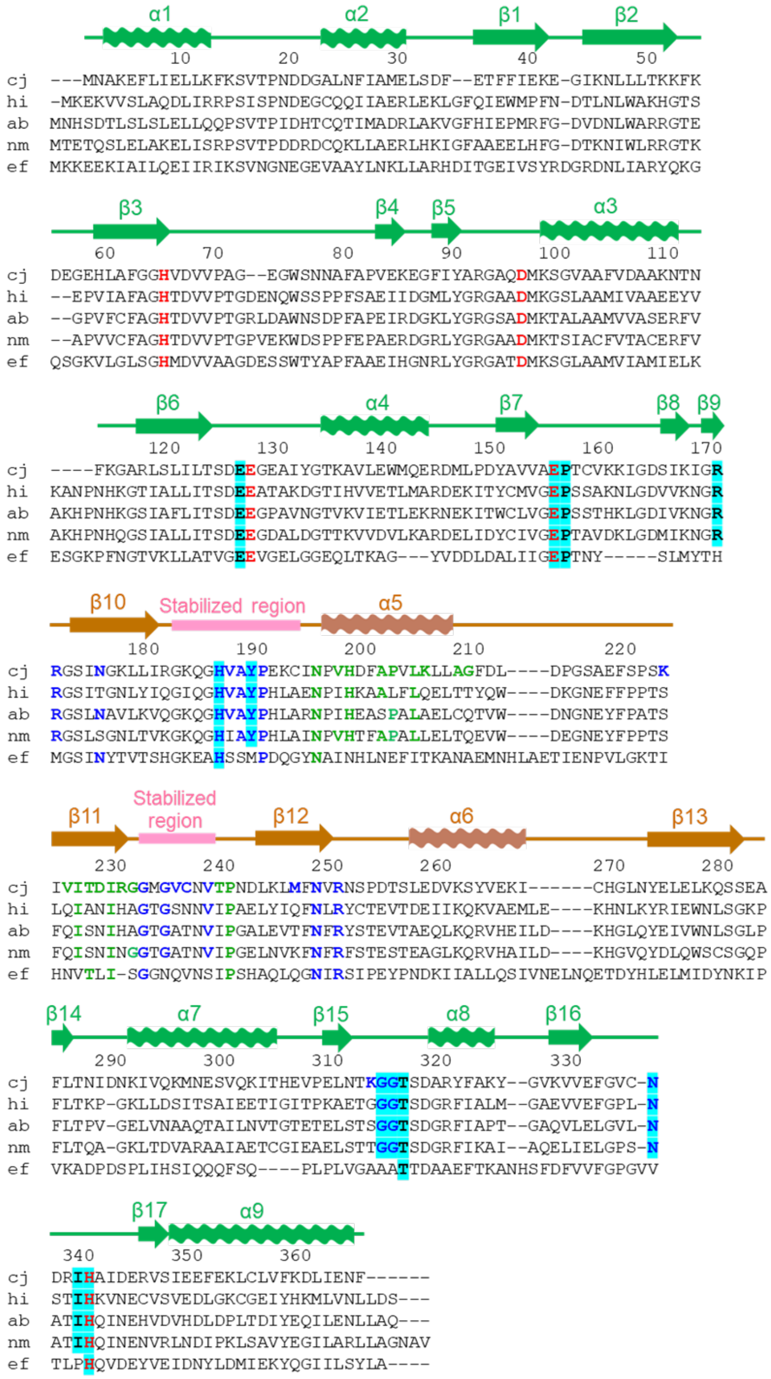

Figure S1. Sequence alignment of cjDapE (cj), hiDapE (hi), abDapE (ab), nmDapE (nm), and efDapE (ef). The secondary structural elements of cjDapE observed in the cjDapE$_{Zn}$ structure are shown above the cjDapE sequence as waves (α-helices) and arrows (β-strands). In the cjDapE sequence, Zn^{2+}-coordinating residues and dimerization interface residues are colored red and green/blue (green, dimerization interface residues observed in both the cjDapE$_{Zn}$ and cjDapE$_{Capto}$ structures; blue, dimerization interface residues observed only in the cjDapE$_{Capto}$ structure), respectively, and captopril-binding residues are highlighted with a cyan background. When these cjDapE residues are conserved as the identical residue types in the orthologs, the corresponding residues are colored or highlighted identically.

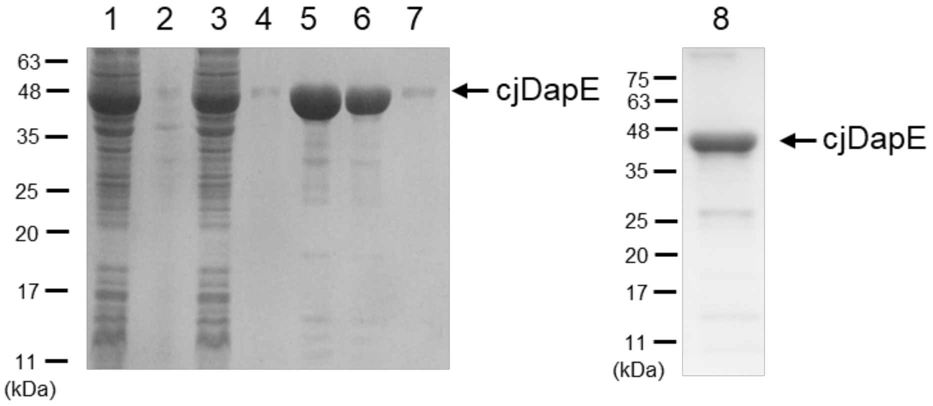

Figure S2. SDS-PAGE analysis of the cjDapE protein obtained after expression and purification. Lanes 1 and 2 show the soluble and insoluble fractions of the cell lysate, respectively. Lanes 3-7 represent fractions from Ni-NTA affinity chromatography (lane 3, flow-through; lane 4, wash; lane 5, 50 mM imidazole elution; lane 6, 100 mM imidazole elution; lane 7, 250 mM imidazole elution). The final purified cjDapE protein obtained after ion-exchange chromatography is shown in lane 8.

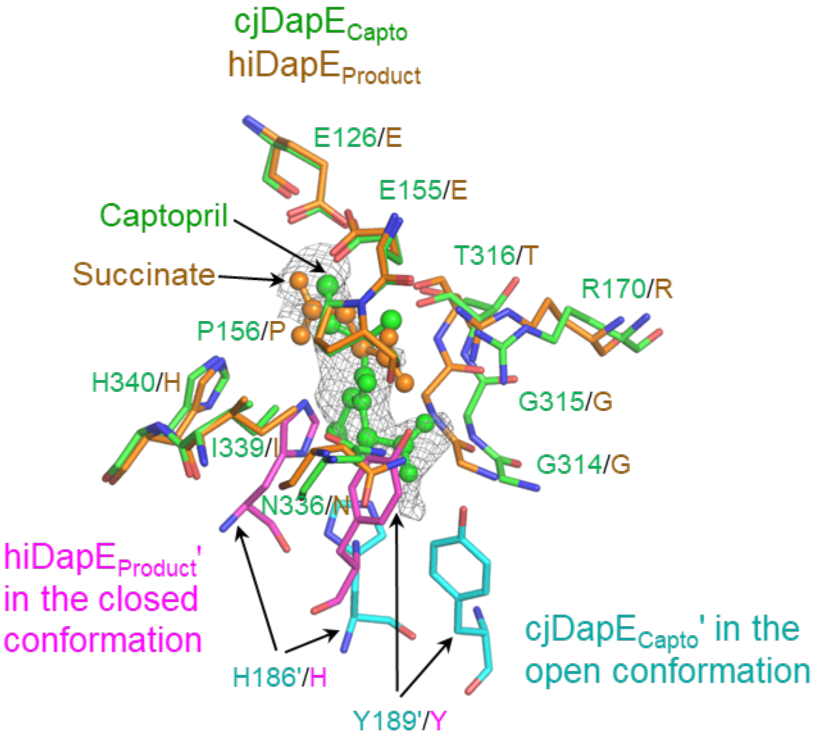

Figure S3. Conservation of residue types between captopril-binding residues in cjDapE and product-binding residues in hiDapE. The hiDapE$_{Product}$ dimer structure (PDB ID 5VO3; hiDapE, orange or magenta sticks; succinate product, orange ball-and-stick model) is superimposed on the cjDapE$_{Capto}$ dimer structure (cjDapE, green or cyan sticks; captopril, green ball-and-stick model) based on one CD. The Fo-Fc omit map for the captopril molecule in the cjDapE$_{Capto}$ structure is displayed as gray mesh at the 2.0σ contour level. The captopril-binding residues of cjDapE are shown as green and cyan sticks for chains cjDapE$_{Capto}$ and cjDapE$_{Capto}$', respectively, and labeled in corresponding colors before the slash. The succinate product-binding residues of hiDapE are displayed as orange and magenta sticks for chains hiDapE$_{Product}$ and hiDapE$_{Product}$', respectively, and labeled in corresponding colors after the slash. The captopril-binding cjDapE residues and product-binding hiDapE residues are identical in residue type. Additionally, residues from chains cjDapE$_{Capto}$ and hiDapE$_{Product}$ generally adopt similar conformers and positions. In contrast, the residues from chain cjDapE$_{Capto}$' are displaced relative to those of chain hiDapE$_{Product}$', because of the conformational shift between the two dimer structures.

Table S1. Crystallographic statistics of the cjDapE structures.

	$cjDapE_{Zn}$	$cjDapE_{Capto}$
**<u>Data collection</u>**		
Space group	$P2_12_12_1$	$P2_12_12_1$
Cell parameters (Å)	a = 49.68	a = 49.72
	b = 116.71	b = 117.36
	c = 198.96	c = 197.78
Wavelength (Å)	0.9793	0.9793
Resolution (Å)	30.00-1.95	30.00-2.45
Highest resolution (Å)	1.98-1.95	2.49-2.45
No. unique reflections	83,887 (4,174) [a]	43,311 (1,982) [a]
R_{merge} [b]	0.077 (0.837) [a]	0.156 (1.101) [a]
R_{meas} [c]	0.086 (0.931) [a]	0.174 (1.284) [a]
R_{pim} [d]	0.037 (0.398) [a]	0.075 (0.643) [a]
$CC_{1/2}$ [e]	0.998 (0.700) [a]	0.991 (0.468) [a]
I/sigma(I)	17.0 (1.3) [a]	11.8 (0.7) [a]
Completeness (%)	98.8 (98.6) [a]	99.2 (91.8) [a]
Redundancy	4.7 (4.8) [a]	4.9 (3.2) [a]
**<u>Refinement</u>**		
Resolution (Å)	30.00-1.95	30.00-2.45
No. of reflections (work)	79,578	41,047
No. of reflections (test)	4,253	2,082
R_{work} (%) [f]	18.0	18.2
R_{free} (%) [g]	20.1	22.4
No. atoms		
Protein	5,352	5,585
Zn^{2+}	4	4
Captopril	-	28
Water	209	14
Average B-value ($Å^2$)	36.5	57.3
Protein	36.4	56.8
Zn^{2+}	51.8	113.4
Captopril	-	163.0
Water	37.7	45.5
RMSD bonds (Å)	0.007	0.008
RMSD angles (°)	0.860	0.973
Ramachandran [h] (favored)	97.9%	97.5%
(outliers)	0.0%	0.0%

[a] Numbers in parentheses were calculated from data of the highest resolution shell.

[b] $R_{merge} = \Sigma_{hkl}\Sigma_i \mid I_i(hkl) - \langle I(hkl)\rangle \mid / \Sigma_{hkl}\Sigma_i I_i(hkl)$

[c] $R_{meas} = \Sigma_{hkl}\{N(hkl)/[N(hkl) - 1]\}^{1/2} \Sigma_i \mid I_i(hkl) - \langle I(hkl)\rangle \mid / \Sigma_{hkl}\Sigma_i I_i(hkl)$

[d] $R_{pim} = \Sigma_{hkl}\{1/[N(hkl) - 1]\}^{1/2} \Sigma_i \mid I_i(hkl) - \langle I(hkl)\rangle \mid / \Sigma_{hkl}\Sigma_i I_i(hkl)$

[e] Correlation coefficient between intensities from random half-data sets.

[f] $R_{work} = \Sigma| |F_{obs}|-|F_{calc}| | / \Sigma|F_{obs}|$, where F_{calc} and F_{obs} are the calculated and observed structure factor amplitudes, respectively.

[g] R_{free} = as for R_{work}, except that 5% of the total reflections were selected at random and omitted from refinement.

[h] Calculated using MolProbity (http://molprobity.biochem.duke.edu).
